# Heparin free dialysis in critically sick children using sustained low efficiency dialysis (SLEDD-f): A new hybrid therapy for dialysis in developing world

**DOI:** 10.1371/journal.pone.0195536

**Published:** 2018-04-26

**Authors:** Sidharth Kumar Sethi, Shyam B. Bansal, Anshika Khare, Maninder Dhaliwal, Veena Raghunathan, Nikita Wadhwani, Ashish Nandwani, Dinesh Kumar Yadav, Amit Kumar Mahapatra, Rupesh Raina

**Affiliations:** 1 Kidney Institute, Medanta, The Medicity, Gurgaon, Haryana, India; 2 Northeast Ohio Medical University, Rootstown, Ohio, United States of America; 3 Pediatric Critical Care, Medanta, The Medicity, Gurgaon, Haryana, India; 4 Pediatric Nephrology, Akron Children’s Hospital, Akron, Ohio, United States of America; University of Sao Paulo Medical School, BRAZIL

## Abstract

**Background:**

In critically sick adults, sustained low efficiency dialysis [SLED] appears to be better tolerated hemodynamically and outcomes seem to be comparable to CRRT. However, there is paucity of data in critically sick children. In children, two recent studies from Taiwan (n = 11) and India (n = 68) showed benefits of SLED in critically sick children.

**Aims and objectives:**

The objective of the study was to look at the feasibility and tolerability of sustained low efficiency daily dialysis-filtration [SLEDD-f] in critically sick pediatric patients.

**Material and methods:**

Design: Retrospective study Inclusion criteria: All pediatric patients who had undergone heparin free SLEDD-f from January 2012 to October 2017. Measurements: Data collected included demographic details, vital signs, PRISM III at admission, ventilator parameters (where applicable), number of inotropes, blood gas and electrolytes before, during, and on conclusion of SLED therapy. Technical information was gathered regarding SLEDD-f prescription and complications.

**Results:**

Between 2012–2017, a total of 242 sessions of SLEDD-f were performed on 70 patients, out of which 40 children survived. The median age of patients in years was 12 (range 0.8–17 years), and the median weight was 39 kg (range 8.5–66 kg). The mean PRISM score at admission was 8.77±7.22. SLEDD-f sessions were well tolerated, with marked improvement in fluid status and acidosis. Premature terminations had to be done in 23 (9.5%) of the sessions. There were 21 sessions (8.6%) terminated due to hypotension and 2 sessions (0.8%) terminated due to circuit clotting. Post- SLEDD-f hypocalcemia occurred in 15 sessions (6.2%), post- SLEDD-f hypophosphatemia occurred in 1 session (0.4%), and post- SLEDD-f hypokalemia occurred in 17 sessions (7.0%).

**Conclusions:**

This study is the largest compiled data on pediatric SLEDD-f use in critically ill patients. Our study confirms the feasibility of heparin free SLEDD-f in a larger pediatric population, and even in children weighing <20 kg on inotropic support.

## Introduction

Acute kidney injury (AKI) in critical care is often secondary to sepsis or shock, and children are usually hypotensive with vasopressor support [1, 2]. Standard extra-corporeal renal replacement therapy (RRT) such as conventional intermittent hemodialysis (IHD) in this subgroup of AKI can be dangerous as it is likely to aggravate hypotension and precipitate catastrophic events [1–3]. In most countries, peritoneal dialysis is underused despite advantages such as lower costs, and less need for infrastructure and specialized training. Technical advances (i.e., flexible and cuffed catheters, automatic cycling, and high and continuous flow peritoneal dialysis) have made it an acceptable alternative [4–7]. The International Society for Peritoneal Dialysis (ISPD) firmly recommends that PD is a suitable modality for patients with AKI, especially in developing countries [8]. For a longer and slower but continuous mode of extra-corporeal RRT, continuous veno-venous haemofilatration/dialysis (CVVH/D) with better haemodynamic stability has been preferred in these groups of critically ill children [9–13]. Unfortunately CVVH/D requires sophisticated costly machines and consumables, and is very labor intensive.

Hybrid therapies providing RRT over an extended period but on an intermittent basis (Sustained Low Efficiency Dialysis, i.e. SLED) using the standard HD machines includes the best of two worlds–the slow sustained modality of CVVH/D, ensuring haemodynamic stability, and better biochemical clearance along with the cost effectiveness of conventional IHD. Evidence accumulated in its favor has been primarily among adults, with pediatric literature still limited [9, 13].

SLED first performed for a patient with AKI in 1945 as an alternative modality of RRT instead of CRRT, is a special form of intermittent dialysis with low dialysate and blood flow rates and prolonged duration [14]. In recent studies, SLED appeared to be better tolerated hemodynamically and showed faster normalization of deranged metabolic parameters compared to conventional intermittent hemodialysis. In a recent meta-analysis, in both RCTs and observational studies there were no significant differences in recovery of kidney function, fluid removal, or days in the intensive care unit. Additionally, SLED showed similar biochemical efficacy to CRRT during treatment (serum urea, serum creatinine, and serum phosphate). The finding that SLED was associated with a lower mortality rate relies on data from observational studies, which are potentially subject to allocation or selection bias, making further high-quality RCTs desirable [15].

In children, two recent studies from Taiwan (n = 11) and India (n = 68) showed benefits of SLED in critically sick children [16, 17]. In a recent survey performed by Raina *et al*., it was concluded that SLEDD-f therapy was only available in 20% of the centers of the developed world and in 25% of the centers in the developing world [9]. Sustained low-efficiency daily diafiltration (SLEDD-f), a conceptual and technical hybrid of IHD and CRRT, is an increasingly popular method of renal replacement for adult patients with AKI [16, 17]. Here, we report the largest study of SLEDD-f in critically sick pediatric population.

## Methods

The objective of the study was to investigate the feasibility and tolerability of heparin free sustained low efficiency daily dialysis-filtration [SLEDD-f] in critically sick pediatric patients. SLEDD-f is the first modality in all critically sick children at this center.

### Indications

Decisions to initiate SLEDD-f and duration (6–12 hours) were taken by both the attending pediatric intensivist and nephrologist according to indications, such as oliguria, positive fluid balance, persistent metabolic acidosis, or electrolyte imbalance refractory to other therapy. Percent Fluid overload was calculated as (fluid in-fluid out)/ICU admission weight x 100. Decision to start dialysis in fluid overload was taken combined by nephrologist and intensivist based on degree of fluid overload (>15%) and presence of other complications including oliguria, metabolic acidosis and dyselectrolytemia.

### Inclusion criteria

All pediatric patients who had undergone SLEDD-f from January 2012-October 2017.

### Working definitions

- SLEDD-f was defined as any hemodialysis session with convection; with blood flow rates ≤5ml/kg/minute and dialysate flow rates not more than twice the blood flow rates, and duration ≥6 hours. For children more than 30 kg, blood and dialysate flow rates were 150ml/minute and 300 ml/min. respectively. According to blood flow (Qb) and filtration rate (Qf), total pre-dilution replacement fluid was calculated as (QfxQb)/(Qb-Qf)x60xtreatment duration (hours).
- Definition of AKI was based on AKIN criteria [18].
- Organ dysfunction and percent fluid overload was defined as per the Goldstein *et al* consensus paper [19, 20].
- Oliguria was defined as a urine output less than 0.5 ml/kg/hour.
- Multi-organ dysfunction syndrome (MODS) meant that the primary disease leading to AKI affected at least one organ system other than the kidneys. Definitions for shock or need for inotrope were as per Goldstein *et al* [20].
- Hypokalemia was defined as serum potassium <3.0 mEq/L and hypophosphatemia was defined as requiring need for phosphate supplementation.

#### Premature termination

Was defined as unplanned termination before 75% of the planned duration.

#### Circuit clotting

Was defined as clotting necessitating interruption of dialysis and change of circuit.

- Mortality was assessed at the time of discharge from the intensive care.

#### Machinery

Fresenius 5008S ® machine was used for all sessions. Dialysate composition was calcium 1.75mmol/L, magnesium 0.5mmol/L, glucose 5mmol/L and bicarbonate was 24-28meq/L. Dialysate potassium was 3meq/L.

- **Dialyzers.** Fresenius FX 40 [children body surface area 0.6m^2^]; FX 50 [children body surface area 1m^2^] and FX 60 [children body surface area 1.4m^2^] were used.
- **Vascular access.** The access used was femoral site or internal jugular vein. Standard hemodialysis catheters were used.
- **Ultrafiltration rate.** Initial ultrafiltration rate was kept at 0.5-1ml/kg/hour, and was increased as per the hemodynamic stability of the patient.

### Measurements

Data collected included demographic details, vital signs, ventilator parameters (where applicable), number and dose of inotropes, blood gas and electrolytes before, during, and on conclusion of SLEDD-f therapy. Pediatric Risk of Mortality III (PRISM III) score at time of admission to PICU was also noted. Technical information gathered regarding SLEDD-f prescription included blood flow rate, dialysate flow rate, replacement flow rate prescribed and achieved duration, ultrafiltration volume, heparin dose and complications if any.

### Statistical analysis

All values in figures and tables are expressed as mean ± standard error. Student’s t tests (unpaired, two-tailed) were used for intergroup comparison, with p value <0.05 indicating statistical significance. Analysis was performed with SPSS software.

## Results

### Patient characteristics

From 2012–2017, a total of 242 sessions of SLEDD-f were performed on 70 patients, out of which 40 children survived **[Table 1]**. Between January 2012-June 2016, 22 sessions of SLEDD-f were performed on 10 children, which were not consecutive. Intermittently, children also received SLED without convection during the period 2012-June 2016.

**Table 1:** Baseline characteristics.

Total (n = 70)	Total (n = 70)	Survivors (n = 40)	Non-survivors (n = 30)	p value
**Age (years)** **Median (Range)**	12(0.8–17)	10(0.8–17)	15(1–17)	0.027
**Weight (kgs)** **Median(Range)**	39(8.5–66)	26(10–65)	45(8.5–66)	0.006
**PRISM at SLEDD-f** **Mean±SD**	8.77 ± 7.22	6.44 ± 5.88	12.95 ± 7.53	0.000 (1.13×10^−8^)
**%FO at SLEDD-f** **Mean±SD**	8.11 ± 7.39	8.83 ± 8.34	6.81 ± 5.1	0.04
**Number of inotropes**	1.08±1.06	1.01±0.98	1.32±1.10	0.04
**Oliguria (n)** **(urine output <0.5ml/kg/hour)**	64 (91.4%)	36	28	0.42
**MODS**	52 (74.3%)	33 (82.5%)	30 (100%)	0.03
**Ventilated**	46 (65.7%)	27 (67.5%)	30 (100%)	0.001
**Sepsis**	29 (41.4%)	16 (40%)	22 (73.3%)	0.007
**Fungal sepsis**	10 (14.3%)	8 (20%)	1 (3.3%)	0.82
**Hospital stay (days)** **Median (Range)**	14.5 (1–46)	17 (3–46)	8 (1–19)	0.0006
**Ventilation (hours) Median (Range)**	68 (0–489)	44 (0–489)	152 (14–332)	0.11

After June 2016-October 2017, all sessions of SLEDD-f were consecutively done on next 60 patients (220 sessions). Since June 2016, all representative pediatric ICU patients received SLEDD-f.

The median age of patients in years was 12 (range 0.8–17 years), and the median weight was 39 kg (range 8.5–66 kg). The mean PRISM score at admission was 8.77±7.22. A total of 46 patients (65.7%) were ventilated for a median of 68 hours (range 0–489). Sepsis occurred in 29 patients (41.4%), of which 10 patients (14.3%) had fungal sepsis. MODS occurred in 52 patients (74.3%). The median length of hospital stay was 14.5 days (range of 1–46).

### Indications

*of SLEDD-f*: SLEDD-f was done for combined metabolic acidosis and fluid overload in 134 sessions, persistent metabolic acidosis (pH<7.1) and hyperkalemia (serum potassium>6meq/L) in 34 sessions, persistent fluid overload in 37 sessions, persistent metabolic acidosis only in 16 sessions, combined metabolic acidosis, fluid overload and hyperkalemia in 20 sessions, and hyperammonemia in 1 session.

### Prescription

Blood flow rate was 4.25±0.82 ml/min, and dialysate flow rates were 9.2±6.2 ml/minute. Replacement fluid administration rate was modified to keep filtration fraction less than 30%. Duration planned was 6.8±1.54 hours and achieved was 6.3±1.6 hours.

### Blood priming

All children less than 20kg were dialysed with pediatric blood lines (Fresenius blood tubings = extracorporeal volume 108ml for 5008-S machine). Whenever the extra corporeal blood volume exceeded 10% of total blood volume (n = 17) the circuit was primed with saline / 5% albumin or packed red blood cell (if haemoglobin <7 gm/dl).

### Ultrafiltration rate

The ultrafiltration rate was 8.75±6.75 ml/kg/hr (mean±SD).

#### Toxin reduction

Pre- SLEDD-f urea (mean ± SD) was 141 ± 104 mg/dL and post- SLEDD-f urea (mean ± SD) was 94.3 ± 84.2 mg/dL (p < 0.05). Pre- SLEDD-f creatinine (mean ± SD) was 4.1 ± 2.9 mg/dL and post- SLEDD-f creatinine (mean ± SD) was 2.9 ± 2.5 mg/dL (p < 0.05).

### Hemodynamic stability

Out of 242 sessions, children were on more than 1 inotrope in 154 sessions (63.6%; norepinephrine with another agent), and more than 2 inotropes in 101 sessions (41.7%). The total number of inotropes was 1.08+1.06 [mean+SD] (non survivors 1.01+0.98 and survivors 1.32+1.10 p = 0.04). Mean number of inotropes pre-SLEDD-f was 1.02+0.98 and post-SLEDD-f was 1.03+0.96; p = 0.40. Pre- SLEDD-f blood pressure (mean ± SD) was 86.3 mmHg and post- SLEDD-f blood pressure (mean ± SD) was 83.5 ± 14.7 mmHg with a p value > 0.05, thus indicating no hemodynamic compromise post the dialysis sessions.

### Acidosis and oxygenation

Pre and Post-SLEDD-f metabolic and clinical parameters have been shown in **Table 2**. There was, however, significant improvement in acidosis, as noted by the post- SLEDD-f serum bicarbonate level. Pre- SLEDD-f bicarbonate (mean ± SD) was 19.8 ± 4.7 mEq/L and post- SLEDD-f bicarbonate (mean ± SD) was 21.9 ± 4 mEq/L (p < 0.05). On the other hand, the oxygenation index did not show any statistical improvement; with the pre- SLEDD-f oxygenation index (mean ± SD) being 17.35 ± 14.5 and the post- SLEDD-f oxygenation index (mean ± SD) being 15.45 ± 14.6 (p > 0.05).

**Table 2:** Clinical and biochemical characteristics before and after SLEDD-f.

Parameters	Pre- SLEDD-f	Post- SLEDD-f	p value
**Mean BP (mmHg)**	86.3 ± 14	83.5 ± 14.7	0.06
**Mean number of inotropes**	1.02±0.98	1.03±0.96	0.40
**Bicarbonate (mEq/L)**	19.8 ± 4.7	21.9 ± 4	0.000 (5.52×10^−6^)
**Urea (mg/dL)**	141 ± 104	94.3 ± 84.2	0.000 (3.5×10^−6^)
**Creatinine (mg/dL)**	4.1 ± 2.9	2.9 ± 2.5	0.000 (7.33×10^−5^)
**Oxygenation index; n = 36**	17.35 ± 14.5	15.45 ± 14.6	0.35

### Complications [Table 3]

#### Premature terminations

Premature terminations had to be done in 23 (9.5%) of the sessions. There were 21 sessions (8.6%) terminated due to hypotension and 2 sessions (0.8%) terminated due to circuit clotting.

**Table 3:** Post SLEDD-f complications.

Parameters	Value (n = 242 sessions)
**Premature session termination**	23 (9.5%)
**Sessions terminated due to hypotension**	21 (8.6%)
**Sessions terminated due to circuit clotting**	2 (0.8%)
**Post-SLEDD-f hypocalcemia(sessions; %)**	15 (6.2%)
**Post- SLEDD-f hypophosphatemia (sessions; %)**	1 (0.4%)
**Post- SLEDD-f hypokalemia (sessions; %)**	17(7.0%)

#### Hypocalcemia, hypophosphatemia, and hypokalemia

Post- SLEDD-f hypocalcemia occurred in 15 sessions (6.2%), post- SLEDD-f hypophosphatemia occurred in 1 session (0.4%), and post-SLED hypokalemia occurred in 17 sessions (7.0%).

### Outcomes

#### Renal recovery

Out of the forty children who survived (40/70), ten children with CKD or end stage renal disease being treated in the intensive care for acute deterioration. Rest thirty children all recovered their renal function on follow up (30/40; 75% of survived patients).

#### Mortality

A total of 30 patients died during this study. Those who died had MODS, a higher PRISM score and were ventilated **[Table 1]**.

## Discussion

SLED is a common dialysis modality in critically ill adults in intensive care units. In view of only two paediatric studies, there is a need for more studies in critically ill children to identify feasibility and tolerability of SLED in this unique population. In the first pediatric study known on the effects of SLEDD-f on this population, Lee *et al* reviewed their experience on SLED-f (i.e EDD along with haemodiafiltration) among 14 critically ill children totalling 60 sessions. The standard prescription included blood flow 5 ml/kg/min, dialysate flow 260 ml/min, hemofiltration 35 ml/kg/h for 8–10 h daily delivered by Fresenius 5008 system. The study concluded that in their cohort of sick children, SLED-f provided good hemodynamic tolerance and correction of fluid overload, pH, and electrolyte imbalance. In addition they also showed a significant drop in inflammatory markers such as adiponectin, interleukin 17 A (IL-17A) and IL 16, post SLED-f session. Despite a relatively high PRISM score (16.8±23.3) they reported an overall 28 day survival of 71.4% [16].

Sethi *et al*, from India showed feasibility of SLED in a retrospective record review from January 2010 to June 2016 from four tertiary pediatric nephrology centers in India. During the study period a total of 68 children received 211 sessions of SLED. Fifty-seven patients were ventilated (84%). Most of the patients had one or more organ system involved in addition to renal failure (n = 64; 94%). Heparin free sessions were achievable in 153 sessions (72%). Out of 211 sessions, 148 sessions were on at least one inotrope (70.1%). Intradialytic hypotension or need for inotrope escalation was seen in 31 (15%) sessions but termination of the session for drop in BP was required in only 20 (9%) sessions [17].

The decreased need for anticoagulation with either unfractionated heparin or citrate when compared to CRRT has been identified as a major advantage of SLEDD-f with the postulated benefit of decreased risk of bleeding and its associated decrease in morbidity and mortality. However, a potential risk associated with not using anti-coagulation is circuit clotting. In a study done by Sethi *et al*., a total of 68 critically ill pediatric patients who required renal replacement therapy received 211 sessions of SLEDD-f and of those, 153 sessions (72%) were done completely heparin free [17]. In previous studies, anticoagulants such as unfractionated heparin and/or citrate have been used. In a similar study done by Lee et al., anticoagulation with unfractionated heparin was done in 76.6% of treatments without bleeding complications, with a bolus of 10–20 IU/kg and a maintenance dose of 5–10 IU/kg/hr; adjusted doses of heparin were used if aPTT > 75 seconds, INR >2, activated clotting time > 275, platelet count < 50,000/microL, and/or a significant risk of bleeding was present [16]. Lee *et al*. reported that in 14 treatments without heparin, one episode of circuit clotting (1/60, 1.7%) occurred which ultimately led to premature termination of SLEDD-f treatment [16]. In a study done by Marshall et al. in adults, circuit clotting in the event of SLEDD-f treatment without the use of anticoagulation was reported to be 26% [21]. In contrast, in study by Sethi et al, overall premature terminations had to be done in 27 sessions (13% of all sessions), out of which 7 sessions had to be terminated due to circuit clotting (3.3%) [17]. In a study involving ICU patients on SLEDD-f therapy for a mean duration of 8 hours with a blood flow rate of 200 ml/min and a dialysate flow rate of 350 ml/min, anticoagulation with heparin was used in 35% of treatments and normal saline flushes were used in 65% of treatments; the incidence of filter clotting in SLEDD-f therapy was 18% with heparin use and 29% without the use of anticoagulation [22]. While the incidence of clotting was higher without anticoagulation use, no significant major adverse events were reported. Fiaccadori et al. demonstrated that regional citrate anticoagulation could be used alternatively as during the 807 SLEDD-f sessions in the study, the incidence of circuit failure from clotting was only 2.4% [23, 24].

Additionally, our study differed in the fact that it was more representative of the standard PICU population as we did not exclude children who weighed less than 20 kg, thus demonstrating the feasibility of SLEDD-f in the small pediatric population. In our study, 17 children were less than 20 kg, with the smallest child being 8.5kg. In this study, we analyze the feasibility and tolerability of heparin free SLEDD-f use in critically ill pediatric patients with AKI. There was no statistical difference in mean blood pressure, number of inotropes, and oxygenation index measured before and after the use of SLEDD-f sessions showing the tolerability of SLEDD-f in the sick subset of pediatric patients.

There are several reports supporting a relationship between increased small solute clearance and improved patient outcomes in critically ill population [25–28]. A greater solute clearance can be achieved by modalities with continuous rather than intermittent. Moreover especially in septic population, a facilitated clearance of larger solutes including inflammatory mediators may also be beneficial [25, 29]. The molecular weight of these solutes is above the cut-off for low-flux hemodialysis membranes, and increased convective clearance with suitably porous membranes to maximize their removal may be helpful in these patients [25, 30]. There are reports showing that SLED is at least as efficacious as continuous hemofiltration in modulating endotoxin induced TNF-alpha production [31]. SLEDD-f includes the development of policies and procedures that enable autonomous treatment delivery by ICU nursing personnel, optimization of (diffusive) clearance for small solutes, and increased (convective) clearance for larger solutes by on-line hemodiafiltration with porous membranes.

The cost-efficacy of SLEDD-f treatment is another major advantage when compared to IHD and CRRT [24]. The filter, tubing set, and solutions required for CRRT treatments makes SLED therapy superior in cost effectiveness. The study by Lee et al. reported that hemodiafilter and tubing for CVVH costs $235 whereas for SLEDD-f it only costs $69; additionally, replacement fluid for SLEDD-f was only $8 when compared with CVVH which was $70 because of its more expensive replacement fluid and larger amounts of fluid needed [16]. Thus this makes each day of SLEDD-f treatment a total of $77 per day compared to CRRT, which is an average of $305 per day. In a different study performed by Berbece et al., it was concluded that SLEDD-f treatment provides solute removal that is equivalent in quality to CRRT but at a significantly lower cost; $1431 for SLEDD-f treatments versus $2607 for CRRT treatment with heparin [22]. Our study, which was done completely anticoagulation free, has lower total costs associated with total treatment duration without any major complications. One session of SLEDD-f in our institute costs 125$, while CRRT initiation costs 500$, plus added costs of replacement fluid and citrate.

Complications associated with SLEDD-f treatment were similar to those associated with IHD and CRRT; these include hypotension, filter clotting, hypokalemia, and hypophosphatemia. Some adult studies have shown however that there is no difference in hemodynamic stability between SLEDD-f and CRRT therapy [24]. For the most part, the hypotension was transient and resolved with discontinuation of ultrafiltration, with normal saline boluses, and/or with the use of albumin. To determine statistical significance, changes in mean blood pressure were taken before and after SLEDD-f therapy in our study and the rates of intradialytic hypotension were recorded. All sessions were well tolerated. Cases of hypokalemia and hypophosphatemia have been reported with the use of SLEDD-f therapy; however, these can be easily adjusted with the dialysate concentration and/or with supplementation.

This study has both strengths and weaknesses; this current study is the largest compiled data on pediatric SLEDD-f use in critically ill patients. Our study confirms the feasibility of SLEDD-f in a larger pediatric population and goes even further to demonstrate that it can be safely performed in children less than 20 kg on inotropic support and without the use of anticoagulation. Being a single center, retrospective study is our study’s limitation. The lack of need of anticoagulation can prove to be of extreme importance as this patient population can often have deranged coagulation, which puts them at an increased risk for anticoagulation related complications.
